# Co-Cultures of Mycorrhizal Fungi Do Not Increase Germination and Seedling Development in the Epiphytic Orchid *Dendrobium nobile*

**DOI:** 10.3389/fpls.2020.571426

**Published:** 2020-10-22

**Authors:** Shi-Cheng Shao, Yan Luo, Hans Jacquemyn

**Affiliations:** ^1^Gardening and Horticulture Department, Xishuangbanna Tropical Botanical Garden, Chinese Academy of Sciences, Mengla, China; ^2^Department of Biology, Plant Conservation and Population Biology, KU Leuven, Leuven, Belgium

**Keywords:** orchid mycorrhizal fungi, Serendipitaceae, specificity, symbiotic seed germination, *Tulasnella*

## Abstract

Orchids are highly dependent on mycorrhizal fungi for seed germination and subsequent growth to a seedling as they provide essential carbon, water, and mineral nutrients to developing seeds. Although there is mounting evidence that orchid seeds are often colonized by multiple fungi simultaneously, most *in vitro* germination experiments focus on mycorrhizal monocultures and little is known about how mycorrhizal assemblages affect seed germination and growth of seedlings. In this study, we compared the effects of mycorrhizal monocultures and co-cultures on seed germination and seedling growth of the epiphytic orchid *Dendrobium nobile*. *In situ* baiting was used to isolate mycorrhizal fungi from protocorms for germination experiments. Germination experiments were conducted under two light regimes for 90 days. In total, five fungal strains were isolated from protocorms of *D. nobile*, indicating that the species was not highly specific to its fungal partners. Four strains (JC-01, JC-02, JC-04, and JC-05) belonged to the Serendipitaceae and one (JC-03) to the Tulasnellaceae. *In vitro* germination experiments showed that germination percentages were higher under light-dark conditions than under complete dark conditions, supporting previous findings that light facilitates germination in epiphytic orchids. While all strains were able to induce protocorm formation and growth into the seedling stage, large differences between fungal strains were observed. Co-cultures did not result in significantly higher seed germination percentages and seedling development than monocultures. Taken together, these results demonstrate that effects of fungal assemblages are not predictable from those of component species, and that more work is needed to better understand the role of fungal assemblages determining seed germination and subsequent growth under natural conditions.

## Introduction

As most other plant species, orchids rely on multiple interactions with other organisms to complete their life cycle. The majority of orchids relies on insects for successful pollination and subsequent seed development (Tremblay et al., 2005), while mycorrhizal fungi are needed for seed germination, seedling recruitment, and growth to adult plants (Swarts and Dixon, 2009; Mujica et al., 2018; Rasmussen and Rasmussen, 2018). Because most orchid seeds are very small (microspermy) and lack sufficient resources, orchid seeds critically depend on mycorrhizal fungi for essential carbon, water, and mineral nutrients (Meng et al., 2019b; Yeh et al., 2019). Before they appear aboveground, orchids are completely reliant on fungi, a condition called initial mycoheterotrophy (Merckx et al., 2013; Jacquemyn and Merckx, 2019). Depending on their dependency on fungi, orchids can remain mycoheterotrophic throughout their entire life or become autotrophic. A substantial number of orchids most likely combines both modes of carbon supply and are therefore partially mycoheterotrophic (Gebauer et al., 2016; Jacquemyn et al., 2017; Schiebold et al., 2017, 2018; Jacquemyn and Merckx, 2019).

Because of their critical reliance on mycorrhizal fungi for seed germination, it has been suggested that high mycorrhizal specificity and/or limited availability of compatible fungi have a strong impact on orchid abundance and rarity (McCormick and Jacquemyn, 2014; McCormick et al., 2018). Orchids associating with a limited number of fungi or with fungi that have a restricted distribution (Waud et al., 2016) can be expected to have narrower distribution patterns compared to orchids that associate with a large number of fungi or with fungi that have a broad distribution (Davis et al., 2015; Těšitelová et al., 2015). Better insights into the relationships between orchid seeds and associated fungi may therefore improve our understanding of the various factors that determine the geographic distribution of orchid species and at the same time benefit symbiotic seedling propagation for *in situ* and *ex situ* conservation and reintroduction programs (Fracchia et al., 2014, 2016; Shao et al., 2017; Decruse et al., 2018).

Studies investigating mycorrhizal specificity in orchids commonly isolate fungi associating with the roots of adult plants, while only a limited number of studies has isolated fungi from developing seeds and protocorms (e.g., Selosse et al., 2004; Bidartondo and Read, 2008; Jacquemyn et al., 2011; Těšitelová et al., 2013; Waud et al., 2017; Vogt-Schilb et al., 2020). These studies have shown that germinating seeds may either associate with a subset of fungi found in adult plants (Selosse et al., 2004; Jacquemyn et al., 2011) or with a different subset of fungi (Bidartondo and Read, 2008; Waud et al., 2017). In addition, *in vitro* experiments have shown that fungi isolated from adult roots may stimulate germination *per se*, but they do not necessarily support subsequent seedling development (Rasmussen et al., 2015) and in some cases may even result in seedling mortality (Zettler et al., 1999; Rasmussen, 2002). Recent experiments have also shown that fungi isolated from protocorms usually lead to higher germination percentages and seedling establishment than fungi isolated from the roots of adult plants (Zi et al., 2014; Zhou and Gao, 2016; Huang et al., 2018; Meng et al., 2019a; Shao et al., 2020), suggesting that fungi associating with adult plants are not necessarily the fungi that induce germination.

Most studies on symbiotic seed germination have used pure cultures based on isolations from orchid roots, preferably from single pelotons (Rasmussen, 2002; Khamchatra et al., 2016; Duran-Lopez et al., 2019), and there are surprisingly few studies that have performed germination experiments with co-cultures (Sharma et al., 2003; Wang et al., 2016). However, communities containing more than one mycorrhizal fungus are likely to influence germination and growth into a seedling differently than do monocultures. For example, mycorrhizal partners may differ in quality, and a more diverse sample of the partner community may be more likely to include the most beneficial partner and therefore increase germination success (Batstone et al., 2018). Different mycorrhizal partners may also occupy different niches and specialize on different resources or have different nutrient access abilities. For example, Nurfadilah et al. (2013) showed that different fungi associated with Australian orchids had different nutrient access abilities. As a result, mycorrhizal assemblages may provide higher access to limiting resources and hence increase germination success. Associating with multiple partners may also lead to more consistent returns through time if partner species exhibit different population dynamics or performance trade-offs (Batstone et al., 2018). Some fungi may be able to induce seed germination, whereas others may stimulate growth after germination. Seeds associating with both types of fungi may therefore have a higher probability to reach the seedling stage than seeds that associate with one of the component species.

In this study, we compared the *in vitro* germination success of the epiphytic orchid *Dendrobium nobile* between monocultures and co-cultures of mycorrhizal fungi. Previous research has shown that fungi isolated from protocorms in the field accelerated seed germination of the related *Dendrobium chrysotoxum* (Shao et al., 2020). We therefore first used seed baiting techniques to isolate fungi associating with protocorms and constructed monocultures and co-cultures of these fungi to assess germination success in *in vitro* trials. Specifically, we hypothesized that (1) fungi isolated from protocorms were able to initiate seed germination and support growth to the seedling stage, and (2) that co-cultures of mycorrhizal fungi significantly increased protocorm formation and seedling development. Because previous research has shown that light conditions can have a large impact on seed germination and seedling formation in epiphytic orchids (Zi et al., 2014; Shao et al., 2020), all experiments were conducted under contrasting light conditions.

## Materials and Methods

### Plant Species

**Figure 1:**
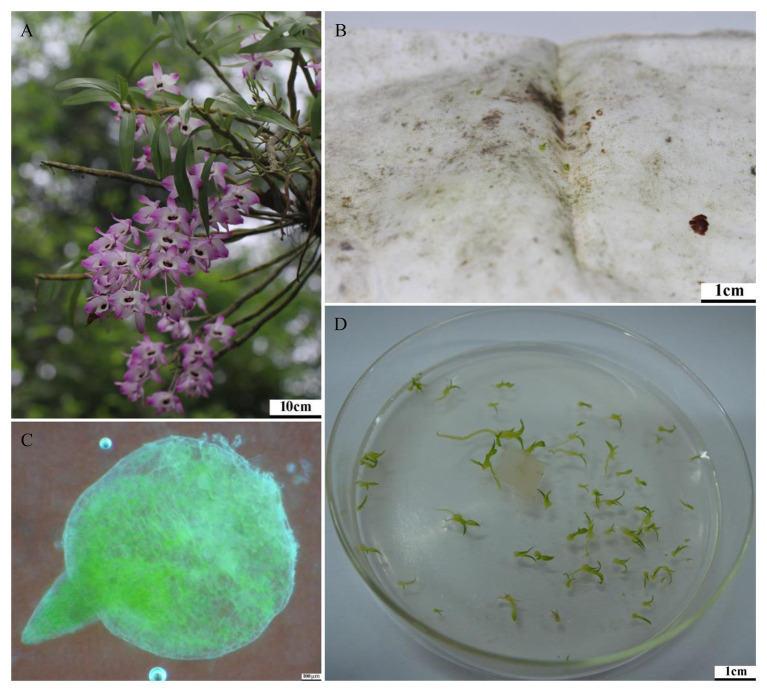
Inflorescence and protocorms induced by *in situ* baiting of *Dendrobium nobile* in Paozhuqing (China). **(A)** Inflorescence of *D. nobile* (Photo by Dr. Liu Qiang); **(B)** *Dendrobium nobile* protocorms induced by modified *in situ* baiting; **(C)** Magnification of protocorm under stereoscope; **(D)** Seedling formation after dual inoculation with fungal strains JC-01 and JC-03.

*Dendrobium nobile* Lindl. (Orchidaceae) is a widely distributed orchid species that occurs in Bhutan, India, Laos, Myanmar, Nepal, northern Thailand, Vietnam, and southern China, including the regions Guangxi, Guizhou, Hainan, Hong Kong, west Hubei, south Sichuan, Taiwan, southeastern Xizang, and Yunnan. The species grows epiphytically on tree trunks or lithophytically on rocks in mountain forests at altitudes between 500 and 1700 m (Chen et al., 2009). The species flowers from April to May (Figure 1A) and fruits are mature the following year in January. According to the China Species Red List (CSRL) and information provided by Gao et al. (2014) and Liu et al. (2015), *D. nobile* is endangered in China (Wang and Xie, 2004). Decline of the species is mainly the result of habitat loss and degradation, limited recruitment in existing populations, and over-collection of the species for medicinal and horticultural purposes (Wang et al., 2011).

### Seed Collection and *in situ* Baiting

Mature and undehisced fruits were collected from a *D. nobile* population growing in Shangjiang town (Lushui county, Nujiang Lisu Autonomous Prefecture in Yunnan) in January 2015. Collected fruits were sterilized with 75% ethanol and washed three times with aseptic distilled water. All seeds were dried using anhydrous calcium chloride (CaCl_2_) in airtight glass containers for 4 days. A part of the seeds was stored in a freezer at −20°C for long-term storage and another part at 4°C for short-term preservation. Based on a subset of 300 seeds, a tetrazolium test (TTC) showed that ~80% of the seeds was viable, i.e., contained a viable embryo.

*In situ* baiting trials were conducted close to natural populations of *D. nobile* in the tropical seasonal rainforests near the village Paozhuqing (Guanlei town, Mengla county, Xishuangbanna Dai Autonomous Prefecture, Yunnan province) in China. The seed suspension consisted of 0.1% sterilized agar and the seeds were homogenized and dispensed into ~2 × 3 cm nylon mesh packets with 45 μm pores, which allows the fungal hyphae to enter the seed packets and to keep the seeds inside the packet (Rasmussen and Whigham, 1993). Every packet contained ca. 300 seeds. In total, 200 seed packets were distributed across three sections corresponding to three naturally growing populations of *D. nobile*. All packets were fixed by plastic wraps on the trees to maintain humidity and avoid desiccation. *In situ* baiting assays were established in January 2015, and the germination process was monitored every month. Once seeds had germinated and developed into protocorms or seedlings, all packets were retrieved in September 2015 and transported to the laboratory for fungal isolation.

### Symbiotic Fungal Isolation From Protocorms

Protocorms (Figure 1B) or seedlings that were retrieved from the seed packets were washed in distilled aseptic water, surface sterilized for 1 min in 75% ethanol and with a NaClO solution with 1% available chlorine for 3–5 min. Subsequently, they were washed three times in sterile distilled water. The protocorms (Figure 1C) were cut into two sections and placed on petri dishes with potato dextrose agar (PDA) and then incubated at 25.0 ± 1.0°C in a fungal incubator. A complete protocorm was cultured to test sterilization. Once the fungal mycelium grew out of the cut protocorm tissue after 3–5 days, the tips were excised and moved to fresh PDA. Pure fungal strains were obtained after repeating the same procedure 3–4 times with the same subculture.

### Molecular Identification of Fungal Isolates and Phylogenetic Analysis

Total genomic DNA was extracted from fresh fungal mycelium using TIANamp Bacteria DNA Kit (DP302) according to the manufacturer’s instructions (Biotech, Beijing, China). ITS regions were PCR-amplified using primer pair ITS1 and ITS4 (White et al., 1990). PCR reactions were performed using Applied Biosystems 2720 Thermal cycler (Bio-Rad, Hercules, California, United States) with 50 μl reaction volumes [1.0 μl DNA, 5.0 μl 10 × Buffer, 1.0 μl Taq polymerase, 1.0 μl dNTP (10 mM), 1.5 μl each primer (10.0 μM) and 39.0 μl ddH_2_O]. The PCR reaction consisted of the following program: initial denaturation at 95°C for 5 min, 35 cycles of denaturation at 95°C for 30 s, annealing at 58°C for 30 s, extension at 72°C for 60 s, and final extension at 72°C for 7 min. Amplification products were purified and sequenced bi-directionally using an ABI3730-XL DNA Sequencer (Applied Biosystems, CA, United States) at Shanghai Personal Biotechnology Co., LTD., China. All sequences were blasted in the GenBank database to match isolates to the genus or species level when rDNA-ITS sequence similarity exceeds 95 or 99%, respectively.

Twenty-five related representative sequences belonging to Sebacinales, including Serendipitaceae and Sebacinaceae from GenBank and four newly generated ones were analyzed for phylogeny, using *Thelephora* sp. as an out-group. Eighteen sequences within Tulasnellaceae from GenBank with newly produced sequence of MH500253 were separately analyzed for tulasnelloid phylogeny, and *Dacrymyces* sp. was selected as an out-group. Alignment of nucleotide sequences was performed by Clustal X version 1.81 and then slightly adjusted manually by BioEdit 7.0.0. Phylogenetic analysis was performed using PAUP 4.0 for maximum parsimony (MP) and MrBayes 3.2.1 (Ronquist and Huelsenbeck, 2003) for Bayesian inference (BI). MP analysis in PAUP used a heuristic search strategy with the following settings: gaps as missing data, addition sequences with random option of 1,000 replicates, 1,000 MP bootstrap replicates were completed using heuristic search with the same search parameters as above. Analyses for BI by MrBayes 3.2.1 were run for 10 million generations with the model of HKY + G. Convergence was considered to be reached when average SD of split frequencies was <0.01 and first 25% discarded as burn-in, Markov chain sampled every 1,000 generations. After the trees were summarized, statistical values were obtained using the sump (burnin = 2000) and sumt commands (burnin = 2000). Node support values were determined as bootstrap percentages (BP) in MP analyses and posterior probabilities (PP) in MrBayes, and both consensus trees were displayed using FigTree v1.4.3.

### *In vitro* Seed Germination

*In vitro* seed germination followed the methods of Zi et al. (2014), with some slight modifications. Ca. 150 seeds were sown in each petri dish. After the seed suspension was sprinkled on oat meal agar (OMA), each petri dish was inoculated with one 0.25 cm^3^ piece of fungal inoculum in case when one single fungus was used or with two or three 0.25 cm^3^ plots when two or three fungi were used. A sterile PDA plug without fungus was also placed as control test. The fungal plugs for the dual inoculation or tri-inoculation were placed together at the center of the petri dishes (Figure 1D). Petri dishes containing seeds and fungi and control treatments were randomly cultured in an incubator with a light photoperiod [12/12 h light/dark (L/D)] with 1,800 lx intensity or were maintained in complete darkness both at 25.0 ± 1.0°C. Both fungi isolated from *D. nobile* and a fungus (CY-KM226996.1) obtained from the related *Dendrobium devonianum* were used in the germination experiments. Each fungal and light treatment was repeated six times. All petri dishes were assessed for protocorm development and seedling growth 20, 50, and 90 days after sowing.

Germination success was categorized into three stages and scored as described by (Arditti, 1967) with slight adjustments (Table 1). To avoid the possibility that embryo swelling (stage 1) was the result of water imbibition and not the result of a true association with the fungus (Rafter et al., 2016), only seeds that reached stage 2 or more advanced stages were considered to have truly germinated. Seed germination success was assessed by calculating the number of seeds reaching stages 2 and 3 divided by the number of viable seeds (i.e., protocorm formation) and the number of seeds developing into a seedling divided by the number of viable seeds (i.e., seedling development). All symbiotic seed germination experiments under *in vitro* conditions were performed at the Xishuangbanna Tropical Botanical Garden in Yunnan (XTBG hereafter).

**Table 1:** Developmental stages and features of symbiotic seed germination of *Dendrobium nobile* (modified on the basis of Arditti, 1967).

Seed germination stage	Characteristic description
0	No germination
1	Imbibed seed, swollen and still covered by testa
2	Protocorm formation and development (rupture of testa, appearance of protomeristem = germination)
3	Seedling formation and development (emergence of first leaf and further developed more leaves)

### Statistical Analysis

To test the hypothesis that fungal inoculation, light conditions, and their interaction affected germination success, a generalized linear model was used with the percentage protocorm formation and seedling development as dependent variables. One-way ANOVA was used to see whether the percentage of protocorm formation and seedling development differed between fungal treatments. Significant differences between treatments were assessed using Duncan’s multiple-range test (*p* < 0.05). We also used independent *t*-tests to determine the effect of light conditions on protocorm formation and seedling development. Finally, *post hoc* multiple-comparisons tests were applied between different fungal treatments on the mean percentage of protocorm formation and seedling development to see whether there were significant differences. All statistical analyses were performed using IBM SPSS version 13.0 (IBM Corporation, Armonk, NY, United States).

## Results

### *In situ* Baiting

**Figure 2:**
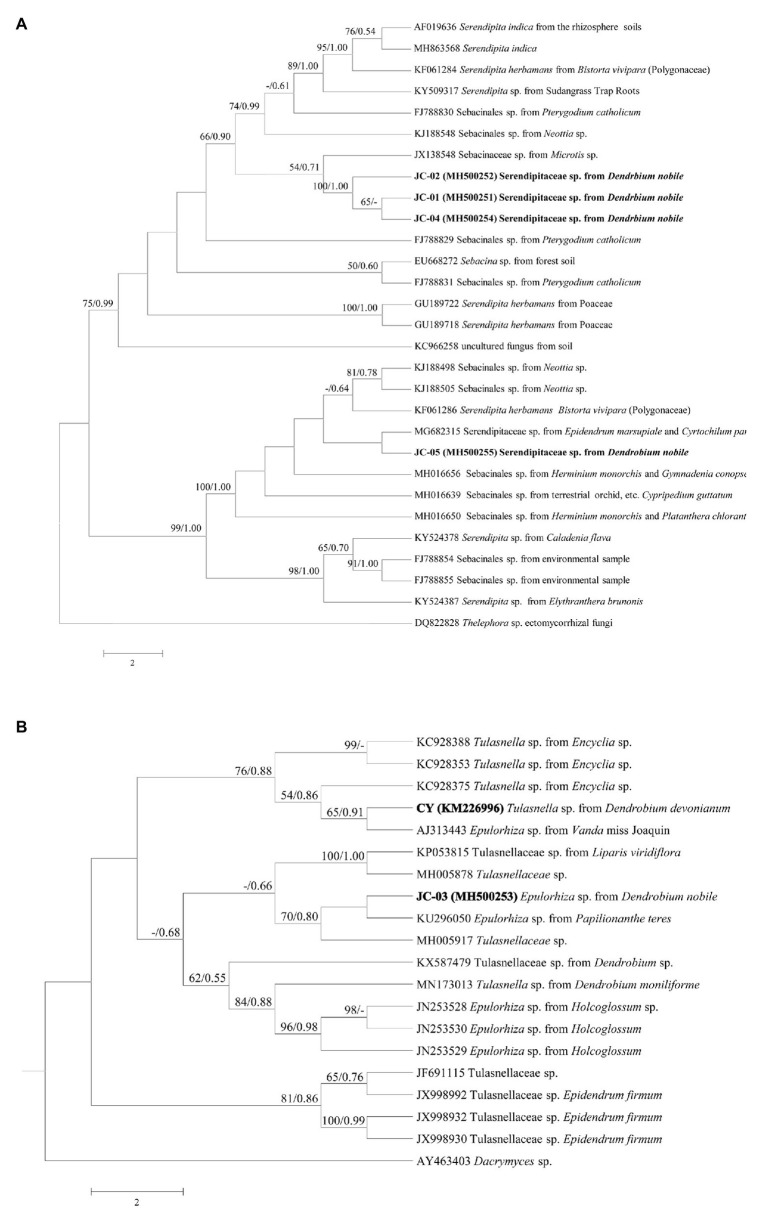
Consensus trees of Sebacinales **(A)** and Tulasnellaceae **(B)** inferred from Maximum parsimony (MP) and Bayesian methods using nrDNA-ITS. Numbers along the nodes are MP and Bayesian inference (BI) support values. Node tips show NCBI accession numbers followed by fungal species name and host species. *Thelephora* sp. in tree A and *Dacrymyces* sp. in tree B were selected as outgroups.

In September 2015, 38 protocorms (Figure 1C) and three seedlings with one leaf were found in 14 out of 96 seed packets (15%), but no seedlings with two leaves were discovered. From these protocorms and seedlings, five fungal strains labeled as JC-01–JC-05 were obtained. Sequences of rDNA-ITS were deposited in GenBank with accession numbers MH500251-MH500255. Blasting in NCBI showed that all retrieved fungi belonged to typical orchid mycorrhizae of Sebacinales and Tulasnellaceae. In the Sebacinales tree, the strains JC-01, JC-02, and JC-04, clustered together with >99% similarity (BP = 100/PP = 1.00) and were close to the Sebacinaceae sp. 5 MB-2012 (JX138548.1; BP = 54/PP = 0.71). The fungus JC-05 was close to the Serendipitaceae sp. (MG682315) with low support values, both of which formed group II (Figure 2A). Only the strain JC-03 identified as *Epulorhiza* sp. (telemorphic genus *Tulasnella*) belonged to Tulasnellaceae tree (Figure 2B). This fungus had the same sequence as *Epulorhiza* sp. Epa-01 (KU296050.1), except for one base pair and formed a subclade with uncultured Tulasnellaceae clone OTU78 (MH005917; BP = 70/PP = 0.80). Fungus CY (KM226996.1), which was obtained from the related *D. devonianum*, was located in the sister clade of JC-03 fungus clade, but with low support values (Figure 2B).

### Symbiotic Seed Germination

**Figure 3:**
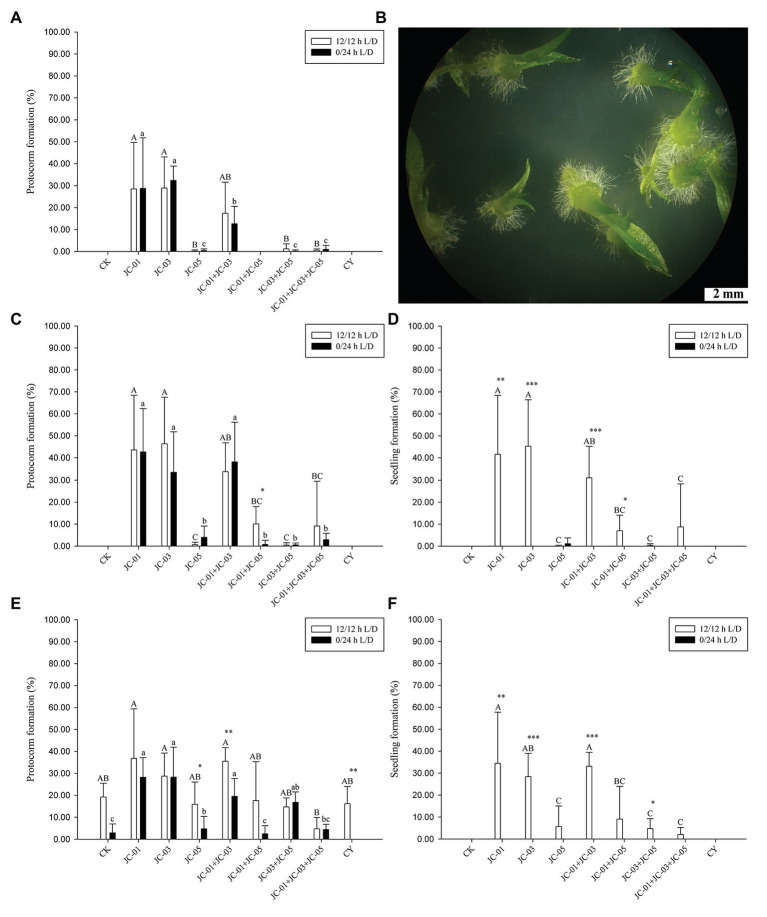
The effects of fungal inoculation and illumination on symbiotic seed germination of *D. nobile* at different times after sowing. The significance among fungal treatments was shown by different capital letter under light photoperiod conditions and lower case letter under dark conditions. Asterisks denote statistical differences between light/dark treatments according to multiple comparisons (^***^p < 0.0001, ^**^p < 0.001, ^*^p < 0.05). **(A)** Protocorm formation at 20 days; **(B)** protocorms and advanced seedlings with leaves and roots at 90 days; **(C)** protocorm formation at 50 days; **(D)** seedling percentage at 50 days; **(E)** protocorm formation at 90 days; **(F)** seedling percentage at 90 days. CK, control test; JC-01: MH500251; JC-03: MH500253; JC-05: MH500255; JC-01 + JC-03: dual inoculation by MH500251 and MH500253; JC-03 + JC-05: dual inoculation by MH500253 and MH500255; JC-01 + JC-03 + JC-05: tri-inoculation by MH500251, MH500253 and MH500255; and CY: KM226996.1.

All fungal isolates from protocorms of *D. nobile* and fungus CY obtained from the related *D. devonianum* were tested for specificity and efficiency on promoting seed germination at 20, 50, and 90 days after sowing. In addition, the following co-cultures were established: JC-01 + JC-03, JC-01 + JC-05, JC-03 + JC-05, JC-01 + JC-03 + JC-05. After 20 days of culturing, protocorm formation was significantly affected by fungal inoculation treatments, but not by light conditions or the interaction between fungal treatment and light (Table 2). Under light/dark conditions, single inoculations using fungal strains JC-01 and JC-03 resulted in the highest percentage of protocorm formation (Figure 3A), followed by the dual inoculation with strains JC-01 and JC-03. Few protocorms were found fungal inoculations involving strain JC-05, JC-03, and JC-05, and JC-01, JC-03, and JC-05 (Figure 3A). No protocorms were formed when seeds were exposed to fungal strain CY and the combination of fungal strains JC-01 and JC-05 (Figure 3A). Under dark conditions, protocorm formation was very similar to that under light conditions: significantly more protocorms were formed when petri dishes were inoculated with fungal strains JC-01 and JC-03 (Figure 3A). No seedlings were produced 20 days after sowing.

**Table 2:** The values presented with factor effect on protocorm formation and seedling development are produced by Generalized Linear Models.

	20 days		50 days		90 days	
Factor (F)	Protocorm	Seedlings	Protocorm	Seedlings	Protocorm	Seedlings
Fungal treatment	23.353^***^	null	24.966^***^	10.071^***^	13.736^***^	11.416^***^
Light	0.007	null	0.875	53.652^***^	24.688^***^	84.698^***^
Fungal treatment x Light	0.148	null	0.608	10.321^***^	1.966	11.416^***^

*** *p* < 0.0001.

Fifty days after sowing, protocorm formation was significantly affected by fungal treatment, but not by light conditions or the interaction between light and fungal treatment (Table 2). Under light conditions, fungal strains JC-01 and JC-03, and the combination JC-01 + JC-03 resulted in the highest percentages of seeds developing into a protocorm (Figure 3C). Fungal treatments JC-05, JC-01 + JC-05, JC-03 + JC-05, and JC-01 + JC-03 + JC-05 resulted in significantly lower number of protocorms (Figure 3C). No protocorms were formed under the control treatment or when seeds were subjected to fungal strain CY (Figure 3C). Similar results were obtained under dark conditions (Figure 3C).

The first seedlings appeared 50 days after sowing (Figure 3D). Both fungal inoculation, light conditions, and the interaction between fungal treatment and light had a significant effect on seedling development (Table 2). Under light conditions, seeds inoculated with fungal strains JC-01, JC-03, and JC-01 + JC-03 developed into significantly more seedlings (45.3 ± 21.1%, 41.6 ± 26.8%, and 31.1 ± 14.1%, respectively) than when seeds were inoculated with strains JC-01 + JC-03 + JC-05 (8.8 ± 19.6%), JC-01 + JC-05 (7.0 ± 7.1%), JC-03 + JC-05 (0.3 ± 0.8%), and JC-05 (0.1 ± 0.3%). No seedlings were formed when seeds were exposed to fungal strain CY and in the control treatment. Under dark conditions, no seedlings were produced except for very few abnormal seedlings when seeds were grown with fungus JC-05 (Figure 3D).

Ninety days after inoculation, fungal treatment and light conditions significantly affected protocorm formation, but the interaction between both factors had no significant impact (Table 2). Protocorms were formed in all fungal treatment test and even in the control (Figure 3E). As before, fungal treatments JC-01, JC-01 + JC-03, and JC-03 resulted in the highest number of protocorms (Figure 3E). Fungal treatment, light conditions and their interaction significantly affected seedling development (Table 2). Seedlings were only observed when seeds were subjected to a light/dark cycle (Figure 3B) and the highest number of seedlings was found when seeds were exposed to strains JC-01 (34.5 ± 23.3%), JC-01 + JC-03 (33.2 ± 6.3%), and JC-03 (28.4 ± 10.6%; Figure 3F). Significantly less seedlings were formed when seeds were exposed to fungal strains JC-05, JC-01 + JC-05, JC-03 + JC-05, and JC-01 + JC-03 + JC-05, and no seedlings were produced in the control treatment or when seeds were grown with fungus CY (Figure 3F).

## Discussion

### Fungi Associated With Protocorms in the Field

In this study, we used *in situ* baiting techniques to isolate mycorrhizal fungi from protocorms of the epiphytic orchid *D. nobile* and to induce seed germination under laboratory conditions. This method has been previously used successfully to induce seed germination of epiphytic orchids under *in vitro* conditions (Wang et al., 2011; Zi et al., 2014; Zhou and Gao, 2016; Shao et al., 2020). Plastic wraps were used to fix the seed packets on tree trunks and to keep the seeds in a humid environment. Our results showed that at least five typical orchid mycorrhizal fungal strains associated with protocorms of *D. nobile*. Four of the five fungi belonged to the family Serendipitaceae within Sebacinales, while the fifth strain belonged to the genus *Epulorhiza* (*Tulasnella*). The strains labeled JC-01, JC-02, and JC-04 shared >99% similarity and therefore could be considered as the same species. These three strains were closely related to a fungal strain that was first isolated in southwestern Australia (Weiss et al., 2016), while the strain JC-03 was located in another subclade. Strain JC-05 was strongly related to a fungus that was previously isolated from protocorms of *Papilionanthe teres* (Zhou and Gao, 2016), suggesting that this strain may occur in a wide range of orchid species.

Interestingly, a previous study has shown that all OTUs found in the roots of adult plants of *D. nobile* belonged to the Tulasnellaceae and that no members of the Sebacinaceae/Serendipitaceae were detected (Xing et al., 2017), even though sample collection took place at the same site. This may suggest that a shift in fungal partners occurs across different life stages. Similar results were reported by Tan et al. (2014), who also obtained two *Tulasnella* strains from the roots of adult plants of *Dendrobium officinale*. However, Chen et al. (2012) isolated three strains of Tulasnellales from protocorms, three strains of Sebacinales and three strains of Cantharellales from roots of *D. nobile*, indicating that the species is capable of associating with fungi belonging to different orders.

### *In vitro* Symbiotic Seed Germination

Compared to the control treatment where no fungus was added, protocorm formation was significantly higher when seeds were exposed to fungal strains JC-01 and JC-03, both under light-dark conditions and under complete darkness. Seedling formation, on the other hand, only occurred under light-dark conditions, and no seedlings were found when seeds were grown under complete darkness. These results support previous findings (Wang et al., 2011; Zi et al., 2014; Huang et al., 2018; Shao et al., 2020) that light, unlike in terrestrial orchids (Rasmussen, 1995), is a prerequisite to induce seedling formation in epiphytic orchids. The highest percentages of seedling formation were observed after 50 days for fungal strains JC-01 and JC-03 (45.26 ± 21.16%, 41.61 ± 26.79%, respectively) under 12/12-h light conditions. Ninety days after seed sowing, all fungal treatments except treatment CY produced seedlings under dark-light conditions, but the number of seedlings largely differed between fungal strains.

All fungal strains that were used in the experiments were isolated from protocorms of *D. nobile* except for the fungus CY (KM226996), which was isolated from protocorms of *D. devonianum*. This fungus has been shown to facilitate seed germination of *D. devonianum* (Huang et al., 2018), but no seedling development was observed when seeds were exposed to fungal strain CY, suggesting strong incompatibility between this fungus and *D. nobile* seeds. Similarly, Wang et al. (2011) isolated fungi from protocorms of *D. chrysanthum* to facilitate seed germination of *D. officinale* and *D. nobile*. These fungi, which all belonged to the Sebacinaceae, were ineffective in facilitating advanced seedling development (5.4%; Wang et al., 2011), also supporting strong specificity for mycorrhizal fungi in *D. nobile*. Similar results have been reported for *Dendrobium aphyllum*, in which only the fungus from protocorms of the host species was able to facilitate seedling formation and development (Zi et al., 2014). Among the fungal strains that were isolated from protocorms of *D. nobile*, there were differences in their ability to induce seedling formation. Both strains JC-01 (Serendipitaceae) and JC-03 (Tulasnellaceae) were able to initiate germination and seedling development with similar efficiency (Figure 3). Although the strains JC-01 and JC-05 both belonged to the family Serendipitaceae and were more closely related to each other than to fungus JC-03, fewer seedlings were formed when seeds were exposed to strain JC-05. To better understand the precise reasons for the observed differences in seed germination and protocorm development, metabolomics and proteomics analyses could be performed to identify the molecules potentially driving interactions between fungi and orchid seeds.

### Co-Cultures vs. Monocultures

Protocorms and seedlings in nature often associate with multiple fungi simultaneously (Bidartondo and Read, 2008; Jacquemyn et al., 2011; Waud et al., 2017), which may suggest that mycorrhizal assemblages enhance seed germination and subsequent seedling establishment. However, the precise role of each fungus in these fungal assemblages is not clear, and it may be that germination is mainly driven by one single fungus and that other fungi play only a role later in the life cycle of the orchid. To test this hypothesis, we created both monocultures and co-cultures of mycorrhizal fungi and compared protocorm formation and seedling formation between them. Our results showed that co-cultures did not increase protocorm formation and seedling establishment, and often led to slightly lower germination percentages than monocultures (Figure 3). In particular, co-cultures involving fungal strain JC-05 (JC-01 + JC-05, JC-03 + JC-05, JC-01 + JC-03 + JC-05) suppressed seedling development and hence significantly fewer seedlings with two leaves were found in these co-cultures compared to monocultures with strains JC-01 and JC-03 treatments. Similar results have been presented by Sharma et al. (2003), who showed that almost all stage 3 and 4 protocorms were found in plates that were inoculated with the key inoculators Bic-70 and Bic-68+70, but significant fewer seedlings of stage 5 were found under co-inoculation of Bic-68+70 compared to inoculation by the single fungus Bic-70. Here, the superior performance of strains JC-01 and JC-03 seemed to be offset when fungal strain JC-05 was added to co-cultures, possibly as a result competition or antagonistic reactions. These results thus indicate that no additive effects were observed and that symbiotic germination experiments therefore are preferentially conducted with single cultures rather than with co-cultures. However, it has to be noted that germination experiments were performed for only 90 days and that it is possible that media that induced lower germination in the end may lead to better development of adult plants (higher number of leaves, roots, etc.). More research across longer time frames is therefore needed to see whether fungal assemblages lead to enhanced performance of developing seedlings and adult plants.

## Conclusion

In this study, we investigated the effect of mycorrhizal assemblages on seed germination and seedling formation in the epiphytic orchid *D. nobile*. Protocorm formation and seedling establishment were compared under two different light conditions between monocultures and co-cultures of fungal strains isolated from protocorms of *D. nobile* in the field. *In situ* baiting using seed packets wrapped in cling film secured ample protocorm formation in the field and allowed successful isolation of mycorrhizal fungi. All fungi were capable of initiating seedling formation, but large differences were observed between fungal strains. Light appeared to be a key factor determining seedling formation, confirming previous results that light is a necessary factor to facilitate seed germination beyond the protocorm stage. The strains JC-01 and JC-03 were most effective in facilitating seedling development and produced the largest numbers of seedlings with two leaves. In addition, these strains initiated seed germination earlier in time than the other strains did. Therefore, both fungal strains show potential for mass seedling propagation and application in conservation and reintroduction programs. Co-cultures of mycorrhizal strains did not increase seedling formation and in some cases even led to lower germination success.

## Data Availability Statement

The datasets presented in this study can be found in online repositories. The names of the repository/repositories and accession number(s) can be found at: https://www.ncbi.nlm.nih.gov/genbank/, MH500251-MH500255.

## Author Contributions

S-CS designed the experiment and performed the field work. S-CS and YL performed *in vitro* seed germination experiments. S-CS and HJ performed the analysis. S-CS wrote the first draft of the paper. All authors contributed to the article and approved the submitted version.

### Conflict of Interest

The authors declare that the research was conducted in the absence of any commercial or financial relationships that could be construed as a potential conflict of interest.

## References

[ref1] Arditti J. (1967). Factors affecting the germination of orchid seeds. Bot. Rev. 33, 1–97. 10.1007/BF02858656

[ref2] Batstone R. T. Carscadden K. A. Afkhami M. E. Frederickson M. E. (2018). Using niche breadth theory to explain generalization in mutualisms. Ecology 99, 1039–1050. 10.1002/ecy.2188, PMID: 29453827

[ref3] Bidartondo M. I. Read D. J. (2008). Fungal specificity bottlenecks during orchid germination and development. Mol. Ecol. 17, 3707–3716. 10.1111/j.1365-294X.2008.03848.x, PMID: 18627452

[ref4] Chen X. Q. Liu Z. J. Zhu G. H. Lang K. Y. Ji Z. H. Luo Y. B. (2009). “Orchidaceae” in Flora of China. eds. Wu Z. Y. Raven P. H. Hong D. Y. (Beijing and St. Louis: Science Press: Beijing and Missouri Botanical Garden Press), 381.

[ref5] Chen J. Wang H. Guo S. X. (2012). Isolation and identification of endophytic and mycorrhizal fungi from seeds and roots of *Dendrobium* (Orchidaceae). Mycorrhiza 22, 297–307. 10.1007/s00572-011-0404-0, PMID: 21779810

[ref6] Davis B. J. Phillips R. D. Wright M. Linde C. C. Dixon K. W. (2015). Continent-wide distribution in mycorrhizal fungi: implications for the biogeography of specialized orchids. Ann. Bot. 116, 413–421. 10.1093/aob/mcv084, PMID: 26105186 PMC4549956

[ref7] Decruse S. W. Neethu R. S. Pradeep N. S. (2018). Seed germination and seedling growth promoted by a Ceratobasidiaceae clone in *Vanda thwaitesii* Hook. f., an endangered orchid species endemic to South Western Ghats, India and Sri Lanka. S. Afr. J. Bot. 116, 222–229. 10.1016/j.sajb.2018.04.002

[ref8] Duran-Lopez M. E. Caroca-Caceres R. Jahreis K. Narvaez-Vera M. Ansaloni R. Cazar M. E. (2019). The micorryzal fungi *Ceratobasidium* sp. and *Sebacina vermifera* promote seed germination and seedling development of the terrestrial orchid *Epidendrum secundum* Jacq. S. Afr. J. Bot. 125, 54–61. 10.1016/j.sajb.2019.06.029

[ref9] Fracchia S. Aranda-Rickert A. Flachsland E. Terada G. Sede S. (2014). Mycorrhizal compatibility and symbiotic reproduction of *Gavilea australis*, an endangered terrestrial orchid from South Patagonia. Mycorrhiza 24, 627–634. 10.1007/s00572-014-0579-2, PMID: 24777596

[ref10] Fracchia S. Aranda-Rickert A. Rothen C. Sede S. (2016). Associated fungi, symbiotic germination and in vitro seedling development of the rare Andean terrestrial orchid *Chloraea riojana*. Flora 224, 106–111. 10.1016/j.flora.2016.07.008

[ref11] Gao J. Y. Liu Q. Yu D. L. (2014). Orchids in Xishuangbanna: Diversity and conservation. Beijing: China Forestry Publishing House.

[ref12] Gebauer G. Preiss K. Gebauer A. C. (2016). Partial mycoheterotrophy is more widespread among orchids than previously assumed. New Phytol. 211, 11–15. 10.1111/nph.13865, PMID: 26832994

[ref13] Huang H. Zi X. M. Lin H. Gao J. Y. (2018). Host-specificity of symbiotic mycorrhizal fungi for enhancing seed germination, protocorm formation and seedling development of over-collected medicinal orchid, *Dendrobium devonianum*. J. Microbiol. 56, 42–48. 10.1007/s12275-018-7225-1, PMID: 29299845

[ref14] Jacquemyn H. Brys R. Cammue B. P. A. Honnay O. Lievens B. (2011). Mycorrhizal associations and reproductive isolation in three closely related *Orchis* species. Ann. Bot. 107, 347–356. 10.1093/aob/mcq248, PMID: 21186239 PMC3043927

[ref15] Jacquemyn H. Merckx V. S. F. T. (2019). Mycorrhizal symbioses and the evolution of trophic modes in plants. J. Ecol. 107, 1567–1581. 10.1111/1365-2745.13165

[ref16] Jacquemyn H. Waud M. Brys R. Lallemand F. Courty P. -E. Robionek A.. (2017). Mycorrhizal associations and trophic modes in coexisting orchids: an ecological continuum between auto- and mixotrophy. Front. Plant Sci. 8:1497. 10.3389/fpls.2017.01497, PMID: 28912791 PMC5583604

[ref17] Khamchatra N. Dixon K. W. Tantiwiwat S. Piapukiew J. (2016). Symbiotic seed germination of an endangered epiphytic slipper orchid, *Paphiopedilum villosum* (Lindl.) Stein. from Thailand. S. Afr. J. Bot. 104, 76–81. 10.1016/j.sajb.2015.11.012

[ref18] Liu Q. Chen J. Corlett R. T. Fan X. L. Yu D. L. Yang H. P.. (2015). Orchid conservation in the biodiversity hotspot of southwestern China. Conserv. Biol. 29, 1563–1572. 10.1111/cobi.12584, PMID: 26372504

[ref19] McCormick M. K. Jacquemyn H. (2014). What constrains the distribution of orchid populations? New Phytol. 202, 392–400. 10.1111/nph.12639

[ref20] McCormick M. K. Whigham D. F. Canchani-Viruet A. (2018). Mycorrhizal fungi affect orchid distribution and population dynamics. New Phytol. 219, 1207–1215. 10.1111/nph.15223, PMID: 29790578

[ref21] Meng Y. Y. Shao S. C. Liu S. J. Gao J. Y. (2019a). Do the fungi associated with roots of adult plants support seed germination? A case study on *Dendrobium exile* (Orchidaceae). Glob. Ecol. Conserv. 17:e00582. 10.1016/j.gecco.2019.e00582

[ref22] Meng Y. Y. Zhang W. L. Selosse M. A. Gao J. Y. (2019b). Are fungi from adult orchid roots the best symbionts at germination? A case study. Mycorrhiza 29, 541–547. 10.1007/s00572-019-00907-0, PMID: 31312918

[ref23] Merckx V. S. F. T. Freudenstein J. V. Kissling J. Christenhusz M. J. M. Stotler R. E. Crandall-Stotler B. (2013). “Taxonomy and classification” in Mycoheterotrophy: The biology of plants living on fungi. ed. Merckx V. (New York, NY: Springer), 19–101.

[ref24] Mujica E. B. Mably J. J. Skarha S. M. Corey L. L. Richardson L. W. Danaher M. W. (2018). A comparision of ghost orchid (*Dendrophylax lindenii*) habitats in Florida and Cuba, with particular reference to seedling recruitment and mycorrhizal fungi. Bot. J. Linn. Soc. 186, 572–586. 10.1093/botlinnean/box106

[ref25] Nurfadilah S. Swarts N. D. Dixon K. W. Lambers H. Merritt D. J. (2013). Variation in nutrient-acquisition patterns by mycorrhizal fungi of rare and common orchids explains diversification in a global biodiversity hotspot. Ann. Bot. 111, 1233–1241. 10.1093/aob/mct064, PMID: 23532043 PMC3662510

[ref26] Rafter M. Yokoya K. Schofield E. J. Zettler L. W. Sarasan V. (2016). Non-specific symbiotic germination of *Cynorkis purpurea* (Thouars) Kraezl., a habitat-specific terrestrial orchid from the central highlands of Madagascar. Mycorrhiza 26, 541–552. 10.1007/s00572-016-0691-6, PMID: 26984810

[ref27] Rasmussen H. N. (1995). Terrestrial orchids, from seed to mycotrophic plant. Cambridge: Cambridge University.

[ref28] Rasmussen H. N. (2002). Recent developments in the study of orchid mycorrhiza. Plant Soil 244, 149–163. 10.1023/a:1020246715436

[ref29] Rasmussen H. N. Dixon K. W. Jersakova J. Tesitelova T. (2015). Germination and seedling establishment in orchids: a complex of requirements. Ann. Bot. 116, 391–402. 10.1093/aob/mcv087, PMID: 26271118 PMC4549959

[ref30] Rasmussen H. N. Rasmussen F. N. (2018). The epiphytic habitat on a living host: reflections on the orchid-tree relationship. Bot. J. Linn. Soc. 186, 456–472. 10.1093/botlinnean/box085

[ref31] Rasmussen H. N. Whigham D. F. (1993). Seed ecology of dust seeds in situ: a new study technique and its application in terrestrial orchids. Am. J. Bot. 80, 1374–1378. 10.1002/j.1537-2197.1993.tb15381.x

[ref32] Ronquist F. Huelsenbeck J. P. (2003). MrBayes 3: bayesian phylogenetic inference under mixed models. Bioinformatics 19, 1572–1574. 10.1093/bioinformatics/btg180, PMID: 12912839

[ref33] Schiebold J. M. I. Bidartondo M. I. Karasch P. Gravendeel B. Gebauer G. (2017). You are what you get from your fungi: nitrogen stable isotope patterns in *Epipactis* species. Ann. Bot. 119, 1085–1095. 10.1093/aob/mcw265, PMID: 28334113 PMC5604585

[ref34] Schiebold J. M. I. Bidartondo M. I. Lenhard F. Makiola A. Gebauer G. (2018). Exploiting mycorrhizas in broad daylight: partial mycoheterotrophy is a common nutritional strategy in meadow orchids. J. Ecol. 106, 168–178. 10.1111/1365-2745.12831

[ref35] Selosse M. -A. Faccio G. Scappaticci G. Bonfante P. (2004). Chlorophyllous and achlorophyllous specimens of *Epipactis microphylla* (Neottieae, Orchidaceae) are associated with ectomycorrhizal septomycetes, including truffles. Microb. Ecol. 47, 416–426. 10.1007/s00248-003-2034-3, PMID: 15107957

[ref36] Shao S. C. Burgess K. S. Cruse-Sanders J. M. Liu Q. Fan X. L. Huang H.. (2017). Using in situ symbiotic seed germination to restore over-collected medicinal orchids in Southwest China. Front. Plant Sci. 8:888. 10.3389/fpls.2017.00888, PMID: 28638388 PMC5461763

[ref37] Shao S. C. Wang Q. X. Beng K. C. Zhao D. K. Jacquemyn H. (2020). Fungi isolated from host protocorms accelerate symbiotic seed germination in an endangered orchid species (*Dendrobium chrysotoxum*) from southern China. Mycorrhzia 30, 529–539. 10.1007/s00572-020-00964-w, PMID: 32562087

[ref39] Sharma J. Zettler L. W. Van Sambeek J. W. Ellersieck M. R. Starbuck C. J. (2003). Symbiotic seed germination and mycorrhizae of federally threatened *Platanthera praeclara* (Orchidaceae). Am. Midl. Nat. 149, 104–120. 10.1674/0003-0031(2003)149[0104:ssgamo]2.0.co;2

[ref40] Swarts N. D. Dixon K. W. (2009). Perspectives on orchid conservation in botanic gardens. Trends Plant Sci. 14, 590–598. 10.1016/j.tplants.2009.07.008, PMID: 19733499

[ref41] Tan X. M. Wang C. L. Chen X. M. Zhou Y. Q. Wang Y. Q. Luo A. X. (2014). In vitro seed germination and seedling growth of an endangered epiphytic orchid, *Dendrobium officinale*, endemic to China using mycorrhizal fungi (*Tulasnella* sp.). Sci. Hortic. 165, 62–68. 10.1016/j.scienta.2013.10.031

[ref42] Těšitelová T. Jersáková J. Roy M. Kubátová B. Těšitel J. Urfus T.. (2013). Ploidy-specific symbiotic interactions: divergence of mycorrhizal fungi between cytotypes of the *Gymnadenia conopsea* group (Orchidaceae). New Phytol. 199, 1022–1033. 10.1111/nph.12348, PMID: 23731358

[ref43] Těšitelová T. Kotilinek M. Jersáková J. Joly F. -X. Kosnar J. Tatarenko I.. (2015). Two widespread green *Neottia species* (Orchidaceae) show mycorrhizal preference for *Sebacinales* in various habitats and ontogenetic stages. Mol. Ecol. 24, 1122–1134. 10.1111/mec.13088, PMID: 25612936

[ref44] Tremblay R. L. Ackerman J. D. Zimmerman J. K. Calvo R. N. (2005). Variation in sexual reproduction in orchids and its evolutionary consequences: a spasmodic journey to diversification. Bot. J. Linn. Soc. 84, 1–54. 10.1111/j.1095-8312.2004.00400.x

[ref45] Vogt-Schilb H. Těšitelová T. Kotilínek M. Sucháček P. Kohout P. Jersáková J. (2020). Altered rhizoctonia assemblages in grasslands on ex-arable land support germination of mycorrhizal generalist, not specialist orchids. New Phytol. 227, 1200–1212. 10.1111/nph.16604, PMID: 32285948

[ref46] Wang H. Fang H. Wang Y. Duan L. Guo S. X. (2011). In situ seed baiting techniques in *Dendrobium officinale* Kimuraet Migo and *Dendrobium nobile* Lindl: the endangered Chinese endemic *Dendrobium* (Orchidaceae). World J. Microbiol. Biotechnol. 27, 2051–2059. 10.1007/s11274-011-0667-9

[ref47] Wang S. Xie Y. (2004). China species red list. Beijing: Higher Education Press.

[ref48] Wang X. Yam T. W. Meng Q. Zhu J. Zhang P. Wu H. (2016). The dual inoculation of endophytic fungi and bacteria promotes seedlings growth in *Dendrobium catenatum* (Orchidaceae) under in vitro culture conditions. Plant Cell Tiss. Org. Cult. 126, 523–531. 10.1007/s11240-016-1021-6

[ref49] Waud M. Brys R. Van Landuyt W. Lievens B. Jacquemyn H. (2017). Mycorrhizal specificity does not limit the distribution of an endangered orchid species. Mol. Ecol. 26, 1687–1701. 10.1111/mec.14014, PMID: 28100022

[ref50] Waud M. Busschaert P. Lievens B. Jacquemyn H. (2016). Specificity and localised distribution of mycorrhizal fungi in the soil may contribute to co-existence of orchid species. Fungal Ecol. 20, 155–165. 10.1016/j.funeco.2015.12.008

[ref51] Weiss M. Waller F. Zuccaro A. Selosse M. -A. (2016). Sebacinales-one thousand and one interactions with land plants. New Phytol. 211, 20–40. 10.1111/nph.13977, PMID: 27193559

[ref52] White T. J. Bruns T. Lee S. Taylor J. (1990). “Amplification and direct sequencing of fungal ribosomal RNA genes for phylogenetics” in PCR protocols: A guide to method and applications. eds. Innis M. A. Gelfand D. H. Sninsky J. J. White T. J. (San Diego: Academic Press), 315–322.

[ref54] Xing X. K. Ma X. T. Men J. Chen Y. H. Guo S. X. (2017). Phylogenetic constrains on mycorrhizal specificity in eight *Dendrobium* (Orchidaceae) species. Sci. China Life Sci. 60, 536–544. 10.1007/s11427-017-9020-1, PMID: 28299575

[ref55] Yeh C. M. Chung K. Liang C. K. Tsai W. C. (2019). New insights into the symbiotic relationship between orchids and fungi. Appl. Sci. 9, 585–598. 10.3390/app9030585

[ref56] Zettler L. W. Burkhead J. C. Marshall J. A. (1999). Use of a mycorrhizal fungus from *Epidendrum conopseum* to germinate seed of *Encyclia tampensis* in vitro. Lindleyana 14, 102–105.

[ref57] Zhou X. Gao J. Y. (2016). Highly compatible Epa-01 strain promotes seed germination and protocorm development of *Papilionanthe teres* (Orchidaceae). Plant Cell Tiss. Org. Cult. 125, 479–493. 10.1007/s11240-016-0964-y

[ref58] Zi X. M. Sheng C. L. Goodale U. M. Shao S. C. Gao J. Y. (2014). In situ seed baiting to isolate germination-enhancing fungi for an epiphytic orchid, *Dendrobium aphyllum* (Orchidaceae). Mycorrhiza 24, 487–499. 10.1007/s00572-014-0565-8, PMID: 24563211

